# Challenges in generating costs and utilisation rates associated with castration-resistant prostate cancer

**DOI:** 10.3402/jmahp.v2.24072

**Published:** 2014-07-04

**Authors:** Siobhan Bourke, Richéal Maria Burns, Caroline Gaynor

**Affiliations:** 1 University of East Anglia, Norwich, UK; 2 University of Oxford, Oxford, UK; 3 National University of Ireland, Galway

**Keywords:** prostate cancer, skeletal-related adverse events, costs, health economics

## Abstract

**Background:**

Prostate cancer (PCa), the most commonly diagnosed cancer among men in the United States and Europe, is an escalating resource allocation issue across healthcare systems in the Western world. The impact of skeletal-related events, associated with castration-resistant prostate cancer (CRPC), is considerable with many new therapies being sought to treat these events in a cost-effective manner.

**Aims:**

The aim of this paper is to provide insight into the level of constraints associated with devising cost frameworks for economic analysis of CRPC in the Irish healthcare setting.

**Methods:**

An informal questionnaire was devised to obtain estimates of utilisation to populate a decision tree model; existing parameters from the literature were also employed. Cost parameters included Irish reference costs, and a costs literature review was undertaken; a healthcare payer perspective was adopted. Pharmacy dosages used for modelling costs were calculated for an average 75 kg male.

**Results:**

The estimated average cost of care associated with adverse events in CRPC was €23,264. Approximately 40% of the costs of CRPC are attributed to skeletal-related events; therefore, reducing the number of skeletal-related events could significantly reduce the cost of care. In attempting to generate accurate and reliable cost parameters, this study highlights the challenges of conducting economic analysis in the Irish healthcare setting.

**Conclusion:**

This study presents leading treatments and associated costs for CRPC patients in the Republic of Ireland (RoI), which are expected to steadily increase with demographic shifts. Further research is warranted in this area due to the limitations encountered in the study.

Prostate cancer (PCa) is seen as an escalating resource allocation issue across healthcare systems in the Western world, mainly due to demographic shifts, increasing incidence and detection, which leads to higher costs (1, 2). It is the most commonly diagnosed cancer among men in the United States and Europe; in the Republic of the Ireland (RoI), PCa accounts for 27.9% of all cancer-afflicted men (2–4). In 2008, it was estimated that there were 258,133 deaths due to PCa worldwide; PCa accounted for 12% of all cancer-related deaths in the RoI. (5, 6). Across Europe, the RoI had the highest incidence of PCa (2006–2010) and the 11th highest mortality rate; incidence is expected to continue to increase attributed to changes in detection practices. (2, 4–7).

Survival rates for PCa are dependent on the stage of diagnosis and the organ concerned; tumours limited to the prostate have a 5-year survival rate of 98%, whereas metastatic cancers have a 5-year survival rate of 32.6% (8, 9). Castration-resistant prostate cancer (CRPC), a late-stage cancer which occurs when there is prostate-specific antigen (PSA) progression, despite consecutive standard hormonal manipulation, is typically a disease of elderly men and is associated with the development of osteoblastic bone metastases (9–11). CRPC metastasis to the bone poses a significant societal burden of cost; bone metastases are associated with severe pain in bone, accompanied by increased bone fragility and can lead to spinal cord compression due to their occurrence in the lumbar spine region as well as the pelvis (12, 13). It is estimated that 80%–92% of men diagnosed with CRPC will have evidence of bone metastasis (14, 15). Major developments have taken place in the treatment of CRPC, which has metastasised to the bone, with biosphosphonates, radiotherapy, and other emerging therapies extending the lives of CRPC sufferers. These therapies attempt to reduce the number of skeletal-related events (SREs), a major component of the cost associated with advanced PCa, as well as to alleviate pain, thus increasing quality of life and survival time (15, 16). The impact of SREs on patients and society is considerable and many new therapies are being sought to treat this adverse event in a cost-effective manner (16–18). Undertaking economic evaluations and cost-effectiveness analysis in the RoI has many constraints; lack of detailed disease-specific reference costs coupled with geographical differences in treatment strategies result in a high degree of uncertainty in determining parameters necessary for cost-effectiveness analysis. The aim of this paper is twofold: to estimate the costs associated with the development of SREs attributable to CRPC and to evidence the level of constraints associated with devising a cost framework for CRPC.

## Materials and methods

### Clinical parameters

A patient algorithm for CRPC was devised from medical literature and previous economic studies in which expert opinions were reported (18, 19). Twenty-two consultants across the main centres of excellence in the West, South and East of the RoI were invited to participate in the study, and the response rate was 18% (n=4). Although the response rate to our invitation to participate in the study was weak, we are confident that this sample of consultants reflect the treatment practice of CRPC in the RoI as two of the consultants treat PCa exclusively in their centres of excellence; given the limited resources and the time constraints of the study further attempts to strengthen the response rate were not possible. All respondents were male and worked in the realm of oncology for the Health Service Executive (HSE); three of the respondents worked in specialist cancer centres managed by the National Cancer Control Program (NCCP). Three of the six HSE regions were represented in the study by the respondents, including HSE West, South and South East, and Dublin/North East. An informal questionnaire was devised to obtain estimates of utilisation to populate a decision tree model; existing parameters from the literature were employed where consensus on treatment patterns could not be achieved. Questionnaires, employing both quantitative and qualitative approaches, ensuing from meetings with experts were, where possible, identical thus not only reducing bias but also providing a platform for discussion around obstacles in identifying consensus on parameters.

The study also sought consultation with a hospital pharmacist to ascertain expert opinion on additional costs associated with delivery of therapy options, adverse events to be included and wastage involved. Through this process, it was established that the weight for estimating dosages would be 75 kg, or 1.8 m^2^. The assumption was made that the patient cohort would have extensive metastases and, therefore, the mean length of survival was deemed 12 months in the model (20). The probability of further treatment after the initial diagnosis of metastatic bone disease is also assumed in the model, as utilisation data were not available for this study.

### Resource utilisation parameters

The study adopted the healthcare payer perspective. Pharmaceutical costs could not be disclosed due to contractual agreement between the hospitals and pharmaceutical companies. Costs of procedures were based on data from HSE Casemix (2012) and patient-level costing at St James's Hospital, Dublin (21). Reference costs of pharmaceuticals were obtained from MIMS (22) and hospital-only pharmaceutical costs (net price) were obtained from the British National Formulary (23), and converted using the appropriate Health Information and Quality Authority (HIQA) guidelines (24). Where costs for a procedure were not available, literature costs were used (8, 12–24). Costs were reported in 2010–2011 Irish Euro. A number of assumptions were made due to lack of consensus and non-availability of data in the RoI. It was also assumed that additional hormone therapy (total androgen blockade) was prescribed for 2 months based on expert opinion (see Table 2). For diagnostic procedures (Table 1) all patients were assumed, in conjunction with expert opinion, to have two of each scan type; this included CT, MRI and X-ray costs.

**Table 1:** Diagnostic procedures: St James Hospital, patient level costing, 2011

Dx procedures	Unit cost (€)	Total cost (€)
CT	80	160
MRI	165	330
X-ray	50	100
Total		590

The length of time the patient received bisphosphonates was estimated to be 3 months. The first-line treatment was chemotherapy comprising six doses. The second-line treatment probabilities were considered in consultation with expert opinion as utilisation data were unavailable (see Table 2). Chemotherapy (second line) was estimated to be limited to three doses, whereas abiraterone acetate was administered for only a month. Additional costs included further complications such as bone pain, spinal cord compression, surgery of the bone and pathological fractures.

**Table 2:** Decision model transition probabilities

Events	Probabilities	Sources
Additional hormone therapy	0.60	Expert Opinion (25)
Skeletal-related events	0.78	Lage et al. (14)
Further complications	0.36	Barlev et al., Lage et al., Felix et al. (12, 14, 26)
Diagnostic procedures	1.00	Expert Opinion (25)
Bisphosphonates	0.80	Nalesnik et al. (27)
Radioisotopes	0.16	Nalesnik et al. (27)
Chemotherapy	0.57	Nalesnik et al. (27)
External beam RT	0.27	Expert Opinion (25)
Second-line chemotherapy	0.20	Expert Opinion (25)
Abiraterone	0.10	Expert Opinion (25)

The cost of palliative care was calculated using information provided by the Irish Hospice Foundation on palliative care by location and was used in conjunction with the Irish Hospice Foundations ‘Audit of End of Life Care in Hospitals in the RoI’ (28). Proportions of PCa patients with metastatic PCa were sourced from the literature and applied to 2011 incidence rates reported by the National Cancer Registry, Ireland (29, 30).

### Modelling methods

A decision model was constructed based on expert advice on the care pathway associated with CRPC using Treeage software. Available costs and outcomes were modelled to ascertain average costs associated with treatment and adverse events of CRPC. Transition probabilities for the decision model are detailed in Table 2 and were sourced from the literature, where available, and expert opinion (12, 14, 25–27).

## Results

**Fig. 1:**
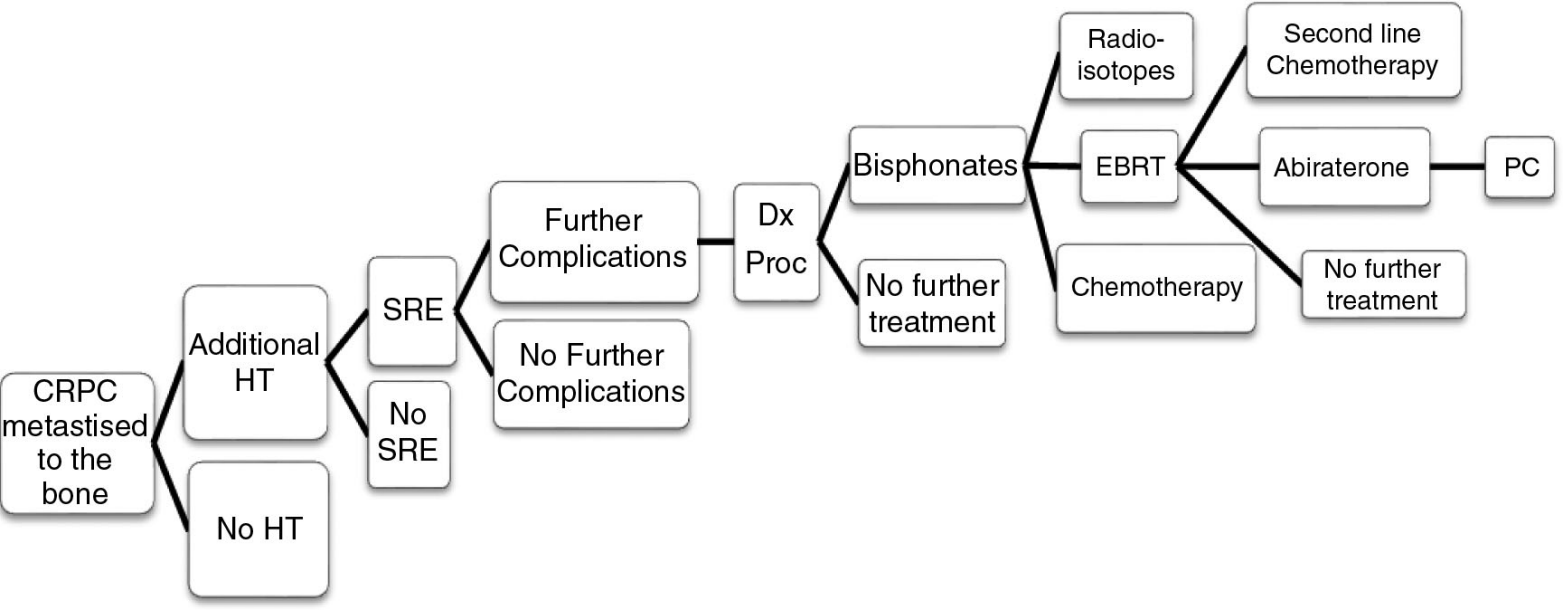
Castration-resistant prostate cancer patient care pathway. HT, hormone therapy; SRE, skeletal-related events; Dx Proc, diagnostic procedures; EBRT, external beam radiation therapy; PC, palliative care.

The analysis estimates that the cost of treating CRPC (metastatic to the bone) in the RoI is €56,889 per patient based on the CRPC patient care pathway algorithm (Fig. 1). Included in this estimate are hospitalisations, medical interventions (such as radiotherapy and chemotherapy), imaging and drug costs; costs of further complications associated with the full treatment pathway were also included (Table 3). Approximately 40% of the costs are attributed to SREs (€23,264 per patient); therefore, reducing the number of SREs could significantly reduce the cost of care.

**Table 3:** Costs associated with adverse events of CRPC

Further complications*	Cost (€)
Hip replacement	12,496
Spinal fusion+deformity (surgical decompression)	37,427
Humerus, tibia, fibula, ankle procedures	5,267
Injury forearm, wrist, foot procedures	7,057
Other musculoskeletal procedures	1,738
Average	12,797

* Conservative estimates reported (no complications).

Source: HSE Casemix, 2012.

Total cost calculations highlight that palliative care accounts for 3% of the total costs, while fractures to the bone account for 16% of the costs. The highest proportion of costs were spent on treatment of bone metastases at 19%; this figure included the yearly cost of external beam radiotherapy, radioisotope treatment and the cost of chemotherapy. The cost of palliative care was estimated per location (Table 4); the most expensive care setting was the hospital, not surprisingly, followed by hospice and long-term care.

**Table 4:** Total cost^a^ of palliative care by location (all costs in €)

Location	Cost (average estimates)	Measure	ALOS (average estimates)	Total cost
Home care	686	Cost per person	13	686
Hospital	1,000	Per day	13	13,000
Hospice	718	Per day	13	9,334
Long-term stay	157	Per day	13	2,043

a Average costs and average length of stay (ALOS) were calculated based on personal communication at Irish Hospice Foundation.

Table 5 highlights the average cost of palliative care per patient. The average cost is €7,193 based on the following assumptions: 1) according to the Irish Hospice Foundation audit paper, between 20% and 30% of CRPC patients receive palliative care; 2) in 2011, there were 2,748 men diagnosed with PCa in the RoI; and 3) based on the literature, it was assumed that 7% of those diagnosed has bone metastases (28–30). Total yearly costs of care vary from €286,299 to €429,488 due to the variation in the estimates of those patients who receive palliative care (Table 5).

**Table 5:** Costs associated with treating patients in palliative care

	Low estimate 20%	Total cost (€)	High estimate 30%	Total cost (€)
Total palliative care patients	40		60	
Hospital	17	248,352	29	372,528
Hospice	2	14,860	2	22,290
Home	9	6,826	15	10,239
Long-term stay	7	16,261	12	24,392
Other	1	N/A	2	N/A
Total cost		286,299		429,448
Average cost*		7,193		7,193

* Rounded estimates reported.

## Discussion

In attempting to generate accurate and reliable cost parameters, this study raises awareness of the challenges of conducting economic analysis in the Irish healthcare setting. Difficulties arose in the collection of utilisation data, as well as consensus on methods employed, given the level of variation in ‘best practice’ across centres of excellence. The records system in the RoI, the hospital in-patients enquiry (HIPE) scheme, does not currently facilitate linkages to patients’ records outside the in-patient setting (31). To capture the treatment pathways accurately for purposes of populating economic models, a more encompassing system tracking all elements of patient care would improve efficiency and therefore provide more robust evidence leading to more informed policy for CRPC. A detailed utilisation database would also monitor and correct for regional disparities in the RoI in terms of resource use as reported in previous works (32, 33).

The average cost of care associated with adverse events in CRPC was €23,264; the relative cost is higher than international comparisons mainly due to improvements in treatment options resulting in longer survival times (8). This paper also raises concerns over the use of reference costs, as these costs are based on diagnosis-related group (DRG) costing, that is, an average package of care for a particular disease group; therefore, it does not allow for assessment of individual cases which lead to estimate bias (33). There is limited insight as to how these reference costs are calculated and what components are included; therefore, it does not allow for transparency when conducting cost analyses for particular disease sub-groups.

Obtaining costs of hospital-only drugs also proved challenging, as each hospital acts as a separate entity in negotiating the price that they pay for drugs associated with the treatment of cancer; therefore, receiving different discounts as a result of variation in negotiation practices. Due to the variation in negotiation across hospitals, an average, representative cost of hospital-administered cancer drugs is difficult to estimate. As these high-cost therapies represent a significant burden of cost to the annual budget for health, it stands to reason that more stringent regulation on utilisation practises and price negotiations by a single government agency would reduce costs and variation in practise and, congruently, provide more transparency for future economic evaluations.

This analysis was subject to multiple limitations; the most obvious limitation is the use of published, bundled complication rates rather than actual rates of utilisation in the RoI. Given the lack of data available, the cost estimates obtained may not accurately reflect the cost of SREs in the RoI. The assumption of the weight of a patient with CRPC could be argued to be too low given the patient demographic and therefore estimated costs may be understated. The use of UK drug costs may not reflect the market in the RoI for pharmaceuticals as trade prices would not be comparable to costs in Irish hospitals; albeit a consensus across Irish hospitals on drug costs is also problematic. DRG average costs are based on a standard package of care; given the lack of national consensus on that package of care for CRPC patients, it is expected that using DRG-related costs for this study introduces a large degree of uncertainty in the estimates. Costs of specific intervention that could not be found in the literature were excluded from the study; these included the cost of ketoconazole and the radioisotope Samarium-153. At the time of this study, denosumab, arguably more potent than bisphosphonates, was not reimbursed in the RoI and so was not included. This study also did not include full drug costs, staff costs, and administration and wastage costs associated with CRPC due to non-availability of data. Finally, the scope of this project provided time and resources to investigate costs associated with the development of SREs in patients with CRPC alone and did not facilitate the cost of treating CRPC in general. However, future research in the area of cost associated with CRPC would allow a more in-depth assessment of the cost differential in treatment of those with and without SREs.

## Conclusion

The estimated financial impact of cancer in the RoI is sizable; PCa in the RoI was recently estimated to amount to €51 million annually (34, 35). Costs associated with adverse events of CRPC have not been examined to date within the Irish context. This study highlights the leading treatments and associated costs for CRPC patients in the RoI, which are expected to steadily increase. Further research is warranted in this area due to high variability in costs and lack of consensus on best practice, which may lead to inefficient and inequitable delivery of care.
